# Reconsidering gender norms in childcare within Chinese migrant families in Portugal

**DOI:** 10.3389/fsoc.2024.1453455

**Published:** 2024-10-09

**Authors:** Yaqun Li, Jean Martin Rabot, Rosalina Pisco Costa

**Affiliations:** ^1^Institute of Social Sciences, University of Minho, Braga, Portugal; ^2^Department of Sociology & CICS.NOVA.UÉvora, University of Évora, Évora, Portugal

**Keywords:** gender roles, childcare, Confucian culture, transnational family, Chinese immigrants, Portugal

## Abstract

Historically, Chinese societies have been characterized by patriarchal structures (Confucianism and filial piety) that assign distinct roles to men and women within the family unit. These norms typically dictate that women take on the primary responsibility for childcare and household duties, while men are expected to be the primary breadwinners. As the authors observe the second generation of Chinese immigrants settling in Portugal, the immigrants grapple with the significant challenge of striving to preserve cultural heritage with the adaptation to Western norms. Therefore, there is a growing recognition of the need to reassess these traditional gender norms. Women in these families are increasingly participating in the workforce, challenging the notion that childcare should be solely to their domain. This study focuses on the experiences of Chinese immigrants, including both married couples and single individuals residing in Portugal. Through qualitative research methods such as interviews (involving 25 participants) and observations, the research aims to gain a nuanced understanding of the attitude and engagement toward childcare, and the ways it differs between men and women in these immigrant families. Participants’ responses indicate that men’s anticipation of women dedicating more time to childcare is notably influenced by educational attainment and financial circumstances. Conversely, women generally believe that being exempt from labor is not a viable alternative irrespective of their educational qualifications. The traditional distribution of caregiving duties does not consistently apply to Chinese female immigrants. By exploring the influence of traditional Confucian culture on migrants’ perspectives and challenges in assuming parental responsibilities, this study highlights gender disparities among Chinese immigrants and propose solutions to address this gender conflict on division.

## 1 Introduction

Gender inequalities in caregiving have long been a prominent and globally recognized issue that has captured the attention of scholars. The vulnerable status of women, particularly mothers, is underscored within the framework of traditional gender roles, where social security predominantly relies on a male breadwinner (Puar, 2012). These gender disparities are especially pronounced across various cultural contexts. In most East Asian societies, family institutions exhibit highly divided gender roles, with mothers primarily shouldering caregiving responsibilities and occupying a less prominent position as earners (Brinton and Oh, 2019). Literature on post-reform China attribute the increased childcare burden on women to specific factors (Zhang, 2017; Du et al., 2019).

Grzywacz and Carlson (2007) argue that achieving work-family balance necessitates mutual understanding and agreement between individuals and their role-related partners in both the work and family spheres. This balance pertains not only to women, but also involves negotiation regarding paid employment and personal life, which encompasses domestic duties share by men and women (Gregory and Milner, 2009). Men and women often express differing feelings about the interplay between work and life. Middle-class women, in particular, highlight their sense of scarification when attempting to achieve a balance, whereas men tend to perceive work–family conflicts as natural (Clark, 2000). This immunity of conflict may mean they cannot understand their partner’s parental concerns (Shirani et al., 2012). A shift toward ‘new fatherhood’ is evident, fathers are more committed to be involved and to nurture their children. In Europe, this new fatherhood is inspired by policy incentives aimed at encouraging greater parental engagement childcare (Heers and Szalma, 2022).

Migrants’ labor participation is usually attached to certain employment characterized by low payment, precarious working environment, and demanding workloads (Gorodzeisky and Semyonov, 2017). For transnational families, low income may make it less likely for children to enroll in formal care (Blossfeld et al., 2017), consequently, creating work–family conflict for immigrant women (Vianello, 2022). Considering this, extended care work is taken on by grandparents, mostly grandmothers (Da, 2003; Du et al., 2019). In short, gender inequality seems to persist across generation. Current literature of Chinese immigrants shows different views. Because some are categorized as ‘middle class’ (Gao and Sacchetto, 2023), most of them do not struggle with financial difficulties. However, gender conflicts among them are inherited from traditional filial culture and the rise of feminism. These immigrants are navigating an acculturation process, both for mothers and fathers. In fact, immigrants often do not fully embrace the viewpoints of their host cultures, while culturally rooted parental roles and norms typically endure without significant alteration (Huang and Lamb, 2015).

The Chinese community makes up a significant proportion of migrants in Portugal. While existing literature has extensively examined aspects such as care support by grandparents (Da, 2003; O’Callaghan et al., 2023), aging caregiving (Bedford and Yeh, 2021; Chow, 2006; Wang, 2020) and parenting (He et al., 2021) among Chinese immigrants in other countries, there remains a scarcity of research focusing on the gender gap in childcare in Portugal. This paper zooms in investigating how Chinese immigrants in Portugal, oriented by filial culture, navigate the gendered division of childcare and aims at exploring the impact of Portuguese culture in such a process. This study specifically addresses three key research questions: (1) How do different categories of Chinese immigrants engage with state-of-the-art childcare practices? (2) What role do traditional values play in shaping gender dynamics in childcare responsibilities? (3) What are the obstructions diminishing parental participation in children’s daily activities?

The paper is structured in three major sections, as follows. It commences with a comprehensive literature review on Chinese culture, particularly on filial piety, Chinese migration process, and gender role disparities related to domestic work. The subsequent section details the methodology adopted in this research. Finally, the paper concludes by presenting and discussing the data and research findings.

## 2 Literature review

### 2.1 Theoretical foundations

Modernization entails the examination of societal shifts entwined with economic progress and industrialization. It acts as a transformative force, influencing the cultural fabric of society (Haviland, 2002). The dissemination of scientific knowledge from the West to traditional societies has led to profound changes in their cultural ethos. Adoption of foreign norms and practices has reshaped family structures, cultural norms, religious frameworks, and societal foundations (Charlton and Andras, 2003). Social and cultural transformations within societies are spurred by the embrace of modern principles by individuals. The increase of women participation in salaried employment is also associated with societal changes (Dioale and Seda, 2001).

Central to society is the institution of family. While traditional societies embraced joint and extended family systems, modern societies have catalyzed the prevalence of nuclear family structures (Plowman et al., 2010). Traditional function of family has gradual modification. The modern economy often displaces individuals from their familial and local roots, channeling their skills into distant job markets, thereby weakening the practice of filial piety. Scholars like Silverstein et al. (2003) as well as Yao (2001) argue that this separation challenges traditional values. Structural-functionalist perspectives suggest that in a modernizing society, the concept of filial piety may lose relevance due to changing roles of elders and extended family structures.

In the western, the theses of family individualization, deinstitutionalization and risk have become powerful explanations for what is now called greater instability, fluidity and liquidity (Bauman, 2000; Beck et al., 1994) in family relationships. These theses argue that the family has lost strength as a social institution, that its bonds are weaker and its normative strength in the sense of conformity is lower. This aspect correlates with the imperative to live one’s “own life” (Beck and Beck-Gernsheim, 2002; Giddens, 1991), which seems to be the predominant script imposed on individuals in late modernity. The theses of family individualization, deinstitutionalization and risk can provide an interesting and fruitful lens for studying filial piety in the context of migration. In China, the rise of individualism has led younger generations to prioritize self-development over traditional priorities like political engagement (Sun and Wang, 2010). While certain values such as family ties and patriotism endure, others like Confucian ethics are on the decline amidst shifting societal norms.

### 2.2 Filial piety and gender norms

China is a nation with a long history of traditional value indicating that childbearing duties are primarily mother obligation (Zhao, 2020; Chan et al., 2012; Ni, 2020; Su, 2014; Xavier, 2017; Yeh et al., 2013). Other East Asian countries (Miao, 2015), primarily referring to Japan (Bornstein, 2017), South Korea (Guo, 2010; Park, 2010) share the similar concept regarding gender roles in childcare arrangements, as all originate from the Confucianism, as giving birth is a normal issue for women who hold traditional concept of gender role within domestic work (Liu and Lummaa, 2019). This gender perception originates from the filial culture. Tracing back through history, “Xiao (孝)” filial piety found its roots in the teachings of Confucius, a staunch advocate of the Zhou order. He believed that through the reshaping of institutions, a sense of social solidarity and harmony can be upheld. The family, known as “jia” (家), was one such fundamental foundational institution. As one of the three most influential schools of thought in ancient China, Confucian emphasis on reverent respect of children toward their patrilineal ancestors and how families were central to human identity and to a family system organized hierarchically (Ebrey, 2003). Consequently, patrilineal system is created. In this system, a family traces its lineage through the male line. It represents the parental authority was established within family, where patriarchy being the rule, and fathers hold powers over children and female family members (Qi, 2014). Filial piety not only stands for a family hierarchy, but also means a filial obligation to care for parents (Blair and Madigan, 2019).

Given the dominant position of Confucianism over the past 2,000 years in China, nature of filial piety that requires obedience, respect, and sacrifice of children to their parents remains among Chinese families. Endeavor to fulfill the wishes of parents shows authoritarian aspect of filial piety. To better understand the parent-children relationship, Yeh and Bedford (2003) in their study introduced dual filial piety model. Instead of a reciprocal relationship, authoritarian filial piety attempts to highlight an intensively compliant relationship between parents and children. In this relationship, other than submission of male offspring to patriarchy stressed, women were expected to defer to male authority within families. Obedient to arranged marriage by parents was considered as being “filial” for women, with a proof that they are subordinate to men’s position on both individual and institutional level.

The entrenched notion that women are exclusively accountable for household chores and raising children, as noted by Cook and Dong (2011), persists within the older generation’s mindset, despite conflicting with the preferences of contemporary Chinese women. Marriage serves is a means to perpetuate the family lineage, a principle elucidated in the *Book of Rites* where marriage was dedicated to venerating ancestors in ancestral temples and securing the future generations’ legacy. While men were restricted to one lawful wife, there existed no constraints on the acquisition of concubines. While having sons may afford women a semblance of authority in their later years, they remain strongly reliant on men for all facets of life and lack autonomy over their own rights (Mathur, 2023). Women are dependent on their husbands and sons in the rest of lives. Even after a husband’s passing, women were expected to adhere to the practice of widow chastity, with remarriage permissible only after a three-year mourning period. However, such remarriages went against tradition, leading society to view these women unfavorably (Mathur, 2023). The groundbreaking New Marriage Law of 1950 furthered the egalitarian principle of free marriage and individual mate selection, liberating women from traditional roles that confined them to domestic duties without autonomy within patrilineal families. During the late 1970s, China’s shift from a planned economy to a market-driven one through “reform and opening-up” brought about increased wealth and educational opportunities. This transformation led to young adults deviating from traditional notions of filial piety, resulting in rising divorce rates, remarriages, and delayed first marriages (Chen et al., 2012; Pochinga, 2004).

Filial piety in differential geographical background shapes different gender norms. China exhibits a significant urban–rural gap in terms of traditional values and production techniques (Wang et al., 2024). In rural regions, residents involve themselves in agricultural activities and uphold patrilineal tradition more staunchly than their urban counterparts, thereby fostering an environment conducive to gender inequality. Other strains of literature also put effort into addressing this gender issue. Zhao’s (2020) research compared the division of childcare between urban and rural regions in China, revealing that in rural areas, gender inequality is deeply entrenched within familial responsibilities, as noted by Li (2004). The research of Yang (2023) has indicated that although decreasing proportion adhering to the traditional gender roles, most of the people still think women should assume childbearing responsibilities. Contrary to such beliefs, researchers like Wang et al. (2024) challenge this notion. Their findings suggest that people’s attitude toward gender inequality does not clearly improve over time, however there is a growing consensus that women should have opportunities for independent careers instead of being confined solely to family duties.

### 2.3 Migration and gender roles attitudes

As a cultural framework, the attitudes of gender roles start to change with the changing of politics, economy and culture (Simpson, 2004; Cano and Hofmeister, 2022). Migration is an international relocation of individuals to a foreign nation, leading to a temporary or permanent settlement (Bartram et al., 2014). Migrating to another country is motivated by various reasons, such as better employment, family reunification (Buttiler et al., 2023; Gao and Sacchetto, 2023), getting married, nicer living environment or looking for an elite education for the children (Czaika and Reinprecht, 2022). During the process of transitioning to a new cultural milieu, the inherent cultural values, especially attitudes toward gender roles from one’s country of origin, can undergo transformation (Harrison and Huntington, 2000). Studies conducted in the United States and Europe have revealed that immigrants hailing from comparatively conservative nations often exhibit a susceptibility to the progressive gender norms prevalent in these more advanced societies (Florian et al., 2022; Röder and Mühlau, 2014; Wang and Coulter, 2019). Notably, second-generation female immigrants display a greater propensity toward embracing an egalitarian approach to gender roles (Wang, 2019). However, immigrants who are from a less developed country, managing for familial conjunction of children to the host country need to redefine their plans for the work and family (Gao and Sacchetto, 2023), particularly immigrants from non-English countries encounter greater challenges of languages barriers after migrating to English speaking countries. Because of the low levels of proficiency in English, making these immigrant parents hard to access to formal care (Sprong and Skopek, 2023), the informal care turns into their final option. This undoubtedly increases the burden on women, often forcing them to prioritize caregiving over their careers.

Despite the rapid modernization and industrialization in China, the enduring value of filial piety remains deeply ingrained in contemporary Chinese society, even among those who have migrated abroad. The cross-border migration of Chinese people dates to the nineteenth century and has involved complex dynamics of family separation, relocation, and transnational extensions. While this historical context is significant, the more recent phenomenon of one-child transnational migration adds a new layer of complexity. A notable trend includes a substantial influx of Chinese students migrating to the United Kingdom for educational and professional opportunities (Liu and Wang, 2020). These individuals often find themselves distanced from extended family members, relying heavily on parental support to maintain familial bonds. Struggling to establish social connections in a foreign land, both within the local community and among fellow Chinese expatriates, these young immigrants are driven by filial obligations to excel academically or marry by a certain age.

It is crucial to recognize that migration represents not just a physical relocation but also a cultural transition, impacting individuals differently based on gender. For instance, Chinese women who immigrate to Western countries like the United States, Canada, and Australia may encounter a return to traditional gender roles, assuming caregiver responsibilities within their families (Ho, 2006; Meares, 2010; Zhou, 2000).

Migration thus shapes not only the geographic journey from one country to another but also the intricate interplay between host and home cultures, especially concerning family dynamics and gender roles. Unequal allocation of childcare and household duties are going through acculturation. In comparison to Chinese women living abroad, their west colleagues admit that exists huge gender bias in workplace, but the major pressure comes from intensive work rather than gender inequality (Runyon, 2021). Research on Chinese migrants in the Canadian context has established that cultural values originating from the migrants’ home country significantly influence the formation of new family dynamics in the host nation (Dion and Dion, 1996; Lain, 1994).

### 2.4 Gaps in gender roles

Women are facing increased work demands entangling traditional gender roles and societal expectations. These factors can have significant implications for their physical and mental well-being (McDonald et al., 2005) and further the life satisfaction (Bola Elegbede and Adebayo Abidogun, 2023; Frone, 2000; Frone et al., 1997; Grzywacz and Bas, 2003; Major et al., 2002). In western society, care work is rather a feminine task for professional caregiver, but also applied within household (Polanen et al., 2017). In families where both partners work full-time, wives generally bear a disproportionate burden of household chores, often shouldering around twice as much household labor as their husbands. Moreover, women tend to handle a larger share of routine and daily tasks within the household (Bianchi et al., 2000). Nevertheless, this unequal distribution of labor is not perceived as unfair by women, who often express greater satisfaction than their husbands while taking on a larger share of household responsibilities (Stevens et al., 2005). The researchers discovered that women’s sense of fairness is more closely linked to their marital relationship than to the division of labor itself.

Although both men and women derive satisfaction from childcare than household labor (Poortman and Van der Lippe, 2009), mothers typically shoulder a disproportionate share of childcare duties compared to fathers, a trend underscored by Bianchi et al. (2000). Particularly mothers often assume the primary parenting role, tending to delegate tasks to fathers rather than fostering shared responsibilities (Craig, 2006; Meteyer and Perry-Jenkins, 2010). We may tend to doubt that having children results in the gender gap, while Giddens’ theory of ‘pure relationship’ (Giddens, 1992) has explained that parenting, which is seldom gender-neutral, tends to worsen disparities in labor division, leisure time, financial resources, and other advantages. The impact of having children on couples stems less from children detracting from their relationship and more from intensifying existing gender inequalities.

Women are still bearing a disproportionate burden of domestic responsibilities and childcare duties (Bola Elegbede and Adebayo Abidogun, 2023). The inequality can be analyzed from two facets. On one hand, it is attributed, to certain extent, to the women’s economic level. Even though women’s labor participation is higher due to liberalizing labor markets, migrants are still disenfranchised and treated unfairly in the workplace (Solinger, 1999), and the wage gap between men and women thus has increased (Maurer-Fazio et al., 1999). To provide more forceful evidence of correlation of women’s income and familial position, it was found that with the support of job and income, women are powerful in decision-making within household (Zhang et al., 2004). On the other hand, some researchers argue that the unequal division of household is associated with men’s educational level. In other words, egalitarian fathers with higher educational level and more stable income demonstrate greater care involvement than ones with traditional conception (Bulanda, 2004).

In the past few years, men’s involvement into domestic work and childbearing has been increasingly advocated for Yang (2023). The reason partly owes to the ‘intensive parenting’ ideology (Lareau, 2011). Through ‘concerted cultivation’ (Vincent and Ball, 2007), parents want to ensure their children turn into responsible citizens (Lister, 2006) and realize optimal development (Fox, 2009). However, several practices prevent fatherhood model to be achieved, therefore they switch to the financial supporters as to demonstrate their involvement (Shirani et al., 2012). Mothers are deemed as primary caregiver and usually fathers do additional help (Verniers et al., 2022). Overall, the ‘intensive parenting’ continually make mothers spend more time with children, the household division remains constantly unequal (Zhao, 2020). It is notable that this ubiquitous gender inequality exists within both transnational and native families (Yeates, 2012).

## 3 Data and methods

### 3.1 Research design

Against this theoretical background and, more specifically, the gender gaps previously identified in the context of the relationship between Chinese migration and filial piety, it seems relevant to develop a study that, from a sociological perspective, allows for an in-depth understanding of the ways in which Chinese immigrants in Portugal, oriented by filial culture, navigate the gendered division of childcare, and the impact of Portuguese culture in such a process. Specifically, the following additional questions seem particularly important to answer: how do different categories of Chinese immigrants engage with state-of-the-art childcare practices; what role do traditional values play in shaping gender dynamics in childcare responsibilities; what are the obstructions diminishing parental participation in children’s daily activities?

To answer these questions, a qualitative research design seems most appropriate, as it seeks to gain a broad and deep understanding of individuals’ experiences and latent meanings (Flick, 2014; Miles and Huberman, 1994). The study involves an in-depth, semi-structured interview, as creating a direct way between researcher and participants (Kvale, 1996; Mason, 2002). The conduction of such interviews aims to gain insights into participants’ inner experiences, offering a depth of understanding beyond what traditional quantitative social surveys can provide.

In line with the qualitative paradigm, and taking into account the research questions, objectives and the literature review, a semi-structured interview was purposefully designed for this research to gain in-depth insights into the experiences of Chinese migrants on related topics. During the interview, a multitude of topics were broached: What are your perspectives on women assuming childbearing responsibilities? Is it essential for women to engage in the workforce? How can conflicts arising from differing opinions on household chore distribution within couples be resolved? In parenting approaches, have you integrated any insights from your parents’ methods into educating your own children? Since relocating to Portugal, which challenges have you faced when engage into childcare? What are your opinions on parenting models of Portuguese culture?

### 3.2 Recruitment and data collection procedures

To obtain a comprehensive understanding of gender roles on childcare among Chinese immigrants in Portugal, most participants hailed from two key immigrant groups respectively: local Chinese^1^ and newcomers.^2^ A total of 25 interviews were conducted with Chinese immigrants sourced from the capital city Lisbon and Oporto. They all either had Portuguese citizenship or permanent resident status and had been living in the country for a minimum 3 years at the time of interview.

Selection criteria centered on these two cities due to their significance. First, these two cities are selected for the large numbers, as being the capital city and second largest city respectively, where assembling the biggest two Chinese immigrants’ communities, Martim Moniz and Vila do Conde. 11 informants are in Oporto, while 14 reside in Lisbon. Note that these two communities are categorized as the major working places for Chinese migrants. More than half of respondents resided in culturally mixed neighborhoods. Second, by gathering insights from these distinct groups, the survey aims to intricate connections between gender values and educational levels among men and women. Last, the geographical diversity across China, such as, Zhejiang province (southern region of China) and Liaoning province (northern region of China), provides valuable insights for readers to examine how regional cultures contribute to shaping distinct gender attitudes.

The interviews were conducted in autumn 2023. Interviews were carried out by the first author through both face-to-face interactions and *WeChat* (the most widely used social media application among Chinese people, with multifunction and convenience) voice calls (usually video calls were rejected by participants). Prior to commencing the interviews, authors obtained participants’ consent either in written form or orally. This consent was securely encrypted, stored, and is scheduled for deletion upon completion of the study. In adherence to privacy measures, all participants were assigned codes for anonymity. For example, male participants were coded as “Mn” (*n* = 1, 2, 3, ..., 13), while female participants were denoted as “Fn” (*n* = 1, 2, 3, ..., 12). Each interview typically lasts 40 min, ranging from a minimum of around 20 min to a maximum of approximately 1.2 h. These interviews were carried out in the mother tongue of the respondents, primarily Chinese Mandarin, then audio recorded, manually transcribed and translated into English.

Participants spanning ages aging 20 to 67 were recruited through direct outreach by the research team and a snowball sampling method. Women above 35 comprised eight individuals, while men’s age distribution was fairly balanced with 7 under and 6 over 35 years old. Of the 25 respondents, 13 men and 12 women were interviewed, with five unmarried men and one unmarried woman. Female immigrant educational levels varied from six with high school or lower education to eight holding a bachelor’s degree or higher. Male immigrants exhibited a similar trend, with seven possessing high school or lower qualifications and the remaining individuals holding degrees or higher education. Nine interviewees hail from various northern regions of China including Liaoning, Shaanxi, Shandong, Xinjiang, and Changchun. Conversely, the 14 interviewees originate from southern areas such as Zhejiang, Fujian, Yunnan, Chongqing, and Shanghai.

All respondents provided valid information on their attitudes toward gender roles in childcare and parenting behaviors, while also offering reflections on how Portuguese culture influences their perception of parenting. One notable facet is that all respondents hold professional positions, whether full-time or part-time, with an average monthly income of 1,500 euros. Nearly all informants indicated they were not facing financial challenges at the time of the interview.

### 3.3 Data analysis

Each transcript underwent a minimum of three times reading enabled authors to be familiarized with the data. A thematic analysis was used to conduct the analysis process, playing an emphasis on exploring aspects of filial culture and gender roles related to childcare rather than theory building (Gray, 2013; Saldana, 2009). The analytical process began with open coding (Charmaz, 2014) whereby codes were applied to the data, for instance, “they (women) should assume the childcare duties” and “both parents should share the childcare duties.” Then, focus coding and constant comparison (Charmaz, 2014) was undertaken. Codes are subsequently used to form categories, for example, “strongly agree upon the filial culture,” “neutral stance on filial culture” and “negatively agree on filial culture.” Data analysis was conducted manually with the support of office applications and services at word processor and spreadsheet level. The first author developed an Excel sheet to record codes, categories and other detailed information, linking them with respondents’ educational attainment, marital condition, financial situation and geographical distribution in China. This approach enhanced the reliability of cross-case analysis when we were facilitating the identification of key theoretical linkages and increased the validity and transparency of the analysis (Deterding and Waters, 2021). At the stage of crosschecking data, noticeably that codes and categories above were allotted based on the previously mentioned migrant groups: local Chinese and newcomers. Furthermore, in this process, authors reassured precise translation, consistent conceptualization and those write-ups reflected respondents’ narratives without imposing our voices on them. By carrying out qualitative analysis, this paper aims to provide a comprehensive and innovative insight into individual’s attitudes toward childcare responsibilities and the impacts of filial piety.

## 4 Findings

### 4.1 Attitudes toward childcare and household work

#### 4.1.1 Chinese traditional values in shaping perception of care work

For unmarried male participants, some of them considered ‘*natural and common*’ that women are expected to assume greater share of childcare responsibilities. Several respondents from Zhejiang province, China, have shared their opinions about it.

*‘Actually, our subconsciousness expects women to dedicate more time to caring duties, particularly towards children, such as spending more time with children. Within my community, there exists a collective understanding that individuals are bound by societal norms as they age. For example, it is commonly accepted that women prioritize caregiving over their careers after giving birth. We all think it is quite normal, and it does not view as unequal among genders (M_2_, 26 years old, Zhejiang).’*

*M_4_ said ‘when envisaging the future with my wife, I hope her taking on a significant role in childcare at home once we have children (24 years old, Zhejiang).’*

The regional cultural context significantly influences these gender dynamics, rooted in traditional values that prioritize women’s central role in child-rearing.

*‘Of course, striking a balance where women manage both a full-time job and family responsibilities is seen as ideal, but it’s often unattainable. Typically, when both partners work, the lack of available support for childcare leads women to prioritize their family over their careers. For me, may be a little boring to do full-time household work. However, if my wife does the care work, it will leave me feeling more energized at work, free from concerns about managing conflicting responsibilities (M_1_, 23 years old, Zhejiang).’*

For a married couple, caregiving is often ‘*automatically*’ assigned to women, likewise, one individual from Zhejiang described:

*‘My wife is able to work primarily due to the additional support from our parents, enabling her to actively engage in the workplace. Without the assistance, she would have had to compromise her career to manage household responsibilities as well (M_8_, 33 years old, Zhejiang).’*

*‘On my mother’s side, she does not pressure her daughter-in-law to take on household or childcare work.’*

Referring to expectation of caregiving from parents, another respondent contemplated the familial relationship between a mother-in-law and daughter-in-law:

*‘We are very lucky residing with my parents, their support is invaluable; they assist us by taking care of tasks like dropping off and picking up my son from school. Despite not pursuing a professional career after my son’s birth, my wife opted for a flexible work schedule as being solely engrossed in household chores did not align with her preferences. My parents have never imposed specific demands on my wife; their approach is one of openness and understanding (M_6,_ 31 years old, Zhejiang).’*

Although the older generation of immigrants may be more accepting of traditional gender roles, a significant number of young male immigrants adhere to gender stereotypes regarding women’s role within the household. However, in contrast, elderly parents instead of favoring to conventional values for women, are the ones who frequently step in to offer care support to young couples, particularly when it comes to aiding daughters-in-law who wish to return to work.

In response, women express differing views compared to men. For instance, a 29-year-old mother shared:

*‘We live together with my parents. Maybe the relationship between mother-in-law and son-in-law is different from the mother-in-law and daughter-in-law, my husband shares a close relationship with my parents, sometimes they are much closer than with me. The support we receive from them is entirely based on their willingness, as we cannot impose on them for assistance. Taking care of most responsibilities falls on me and my husband. With him primarily focused on earning income, I made the decision to step back from my career. Juggling motherhood is no simple task, often leading us to make sacrifices willingly. Thankfully, our earnings are sufficient, and living with my parents allows us to keep expenses low. (F_2_, 29 years old, Fujian).’*

The example demonstrates a prevailing trend where women often willingly choose to leave their jobs to care for their children, while the male interviewees seldom consider this option. Whenever it is available, women are still trying to find even a part-time job. Conversely, one respondent challenges this traditional gender roles:

*‘Everyone needs to work; it is essential to strive for economic independence through work. Opting to be a stay-at-home mom can lead to financial dependence on one’s spouse, which is passive and can diminish a woman’s authority over time. In my opinion, women should purse employment regardless of the income it generates. Even amidst challenges of earning less, seeking additional support like paid-care work can help women maintain their autonomy and self-sufficiency (F_3._ 33 years old, Zhejiang).’*

*‘I prioritize my own well-being above all else, finding it inconceivable to sacrifice my life for another, even for my own children.’*

Despite originating from the same province back in China, women no longer view childbearing as exclusively their primary responsibility; instead, they prioritize their own happiness and autonomy. Similarly, interviewee M_11_ is against gender stereotypes by emphasizing the significance of mental well-being for women from an alternative perspective:

*‘We both manage a Chinese restaurant while juggling our professional commitments. Despite having given birth just one month ago, my wife is able to care for our newborn because the restaurant is not bustling, and we have supportive employees willing to pitch in. Leaving her alone at home would be more exhausting, as without any assistance, she would lack companionship and be more exposed to postpartum depression (M_11,_ 30 years old, Fujian).’*

*‘On the other hand, I respect my wife’s autonomy. If she desires to work, I will not compel her to remain at home.’*

When examining geographical disparity, we often distinguish between northern and southern China. While Zhejiang and Fujian are situated in the southern region, there exist differing attitudes among men regarding gender dynamics. Interestingly, women’s perceptions do not seem to align with regional cultural norms.

#### 4.1.2 Educational level and financial status

Four male respondents from Zhejiang Province who have reflected their attitudes toward gender roles inclined to traditional values, without any exception, owned high school degree or below. Findings from newcomers (as explained in the *Method* Section) expressed their opinions which are different from the local immigrants, particularly male migrants:

*‘Women definitely have to work, it’s a way of expressing their self-worth. If they do not work, then they are completely out of touch with society and do not know what’s popular in the world, and the way people think nowadays, they have to work. Although I can afford the daily expenses of the family, and she may not make much money from the job, but if you do not work and stay at home every day with nothing to do, it will create a lot of family conflicts. At that time, after our child was born, she did not give up her job and also worked while taking care of the child in the shop (M_10_, 62 years old, Changchun, professor at university).’*

*‘Both men and women must engage in employment. Earnings are dependent on individual capabilities, yet staying connected with society necessitates work as people inherently possess social qualities. Interaction with others and acquiring knowledge remain crucial aspects of life.*

*Childcare responsibilities fall on both parents, emphasizing shared contributions rather than mom solely sacrificing her career for family obligations (M_12_, 44 years old, Shanghai, degree in Master).’*

*‘My wife is constantly working. Household chores are a shared responsibility in our family, regardless of gender. We all chip in as needed; when we are busy, we may do less or none at all, but whenever someone has the time, they help out with tasks like cooking, laundry, and cleaning. (M_13_, 55 years old, Xinjiang, degree in Doctor).’*

For female group, most of them value themselves and contribution of women to family and regardless of educational level and original background in China. Many mothers have stated:

*‘In a family, there are no hierarchies based on status; instead, each member must fulfill their roles, maintain a distinct division of household, and offer assistance to one another as needed (F_5_, 36 years old, Liaoning, degree in Master).’*

*‘I am currently a devoted full-time wife. I believe that a wife’s contribution to the family is as valuable as a husband’s financial contribution. Each partner plays a unique role in the division of labor within the household (F_9_, 38 years old, Shanghai, degree in Bachelor).’*

*‘Our family adheres more closely to traditional Chinese cultural gender roles, where women primarily manage household responsibilities while men focus on providing financially. Since my mom works, she takes on tasks like cleaning and caring for the children at home. But I do not agree with this perception, in my own family, my husband and I share the household work (F_15_, 33 years old, Anhui, degree in Bachelor).’*

*‘In our family, we share equality with a distinct labor division; while Dad focuses on long-term vision, I handle the present moment (F_14_, 55 years old, Beijing, degree in Bachelor).’*

Interviewee F_11_ with 37 years old from Zhejiang province still remains single and reflected that:

*‘Men face challenges earning money outside, while women encounter difficulties managing household chores and caring for children. Thus, everyone contributes equally to the household (graduated from middle school).’*

We can see that education level significantly influences men’s attitudes toward gender stereotypes, as evident from their responses. Shifting focus to female respondents, despite varying educational backgrounds, they collectively emphasize the significance of women’s contributions to caregiving and household responsibilities. While prioritizing happiness and advocating for dignity, many women persist in challenging societal norms.

Education level plays a pivotal role in shaping men’s attitudes toward gender stereotypes, a fact underscored by their responses. When considering female perspectives, it becomes apparent that regardless of diverse educational backgrounds, women consistently highlight the importance of women’s roles in caregiving and household duties compared to men’s economic contribution to the family. In their pursuit of happiness and dignity, numerous women bravely confront societal expectations, striving to challenge entrenched norms.

#### 4.1.3 Effective communication and mutual respect in the division of household work

In this section, data was explored having as focus the ways how gender-based conflicts over household chores can be resolved. In the case of dual income married couples, an understanding can be reached regarding the division of household responsibilities, albeit not necessarily in equal parts. As F_2_ indicated:

*‘Since both my parents work, there is not a set person assigned to household tasks. We take turns based on availability. If my mom is free, she takes on the responsibilities; if not, then my father steps in. This same dynamic extends to my father-in-law, who actively participates in housework, influencing my husband and me. We believe that chores should come from a place of personal choice rather than obligation. After long workdays, neither of us desires to clean. Therefore, whenever one of us has some free time, we take on the cleaning duties (29 years old, Fujian).’*

Among the interviewees, growing up in an open and egalitarian family setting can influence a boy’s perspective on gender norms. M_5_ shared that in his family, his parents handle the household responsibilities. He envisions a partnership where his significant other is free to opt out of chores if feasible. He is open to supporting her pursuits outside the home, including a career. Alternatively, if his spouse prefers to focus on homemaking, he appreciates her dedication to managing household affairs. Ultimately, he values her autonomy and believes in letting her make her own choices without coercion. The primary trait he seeks in a partner is positivity.

Data points to the existence of a culture in Wenzhou city (belongs to Zhejiang) that seems to foster a culture that promotes gender equality on household responsibilities. The evident distinction is between household division and childcare. 15 of male respondents express their agreement on sharing household duties with their partners regardless of educational level or geographical background in China. While four male interviewees regarded childcare work as women’s primary task.

Furthermore, information offered by married women and single ladies has emphasized the importance of respect in dealing with disagreements over division and in maintaining a harmonious family atmosphere.

### 4.2 Parental roles in Portuguese society

Numerous factors exert effects on performing specific tasks within realm of childcare among fathers and mothers. In this section, we will explore two key elements that are commonly observed among Chinese married couples that prevent parents from actively engaging in their children’s care activities.

#### 4.2.1 Language barriers

Overcoming language barriers is likely a shared aspiration among first-generation immigrants. In interviews, 12 informants highlighted that either their parents or the younger immigrants who have resided in Portugal for several years, still struggle to meet the linguistic proficiency necessary to effectively use Portuguese in both their professional and everyday lives.

*‘My parents migrated to Portugal nearly 20 years ago. Actually, I was separated from my parents until turned 11 years old. I have an older sister who lived with my parents in Portugal all the time. They (my parents) once explained their choice of locating me with my grandparents in China. Although they have a shop selling* var*ious products to the local Portuguese people, their language proficiency barely allows them to engage in easy conversation with the Portuguese regarding the topic on price, product quality or anything else. They are unable to attend parent-teacher meetings at my sister’s school or consult a doctor. Many of the time, my sister takes on the role of translating Portuguese into Chinese. Therefore, it was not allowed for me to live with them (M_3_, 21 years old, university student, Chongqing).’*

Aside from older immigrants, newcomers also face the same problem.

*‘We are both not good at Portuguese or English. It is essential to learn the language because it is useful in our daily life. In addition to communicating with guests, we are planning to send our child to an institutional nursery when she reaches 4- months old. In that case, we will better understand what the caregivers are saying. Since my wife and I will not have much time to care for the baby, paid care is the best option (M_11_, 30 years old, running a restaurant, Fujian).’*

*‘I am alone in Portugal taking charge of my son’s everyday life and his education. My wife is in China to take care of our aging parents. While my English is just all right, I can communicate with teachers in English. However, since Portuguese is the official language, it is still necessary to master it. To be honest, I realized that language fluency is crucial for parental involvement. If one parent has a better understanding of Portuguese or even English, then he/she may naturally tend to assume more responsibilities (M_12_, 44 years old, Shanghai).’*

F_7_, a devoted full-time mother, shared that her family temporarily relocated to Portugal due to work commitments. Her husband is employed by a Chinese company as a technician and speaks Mandarin exclusively. In light of this, she has taken on the responsibility of managing their child’s education.

*‘After moving to Portugal, I picked up Portuguese, even though I am fluent in English. Despite his strong willingness to engage, my daughter’s father struggles to participate actively in her educational pursuits, especially when he is away on business. Balancing responsibilities, we have differing parenting styles but have discussed and found a harmonious compromise that neither of us finds unfair. Previously employed in Shanghai, a cosmopolitan hub, I can attest that being a full-time mother is more challenging than one might realize these days (F_7_, 49 years old, Shandong).’*

Data has indicated that a lack of proficiency in the local language prevents Chinese migrant parents from enrolling their children in formal education or care services, forcing them to balance work and childcare responsibilities.

#### 4.2.2 Financial instability

Contrary to newcomers, local Chinese immigrants highlighted the challenges they face in caring for their children. They explained that as business owners, both partners are deeply involved in various operational tasks such as stock replenishment, categorization, arrangement, and financial calculations. When one partner is absent from work, the workload significantly increases for the other. In the absence of extended caregiving options or paid care services, many business owners bring their children to the shop, though this practice falls short of being considered proper ‘parenting’. One father shared:

*‘Our dedication to work is undeniable, driven by both diligence and the pursuit of financial stability. While we acknowledge the limitations of upward mobility through this path, our business is our lifeline. We do not rely on state welfare; our success is entirely dependent on our own efforts. The relentless nature of our industry means that working less equates to earning less, making hard work a constant reality. Consequently, our time with our children is limited, and having them accompany us at the shop is often the best solution (M_8_, 33 years old, Zhejiang).’*

M_4_ who is unmarried demonstrated his opinions:

*‘Most of Chinese immigrants I know lead a repetitive life: toiling on weekends, moving from one shop to another as each one closes, in a ceaseless cycle of work. Contrasting this, Portuguese parents prioritize their children’s mental-health and development. They take children to parks, to beach, to exercise, dedicating significant time by the children’s side. Reflecting on this, I cannot help but feel a tinge of envy, as my own childhood was confined to the familiar routine of home, school, and the family shop. This divergence leads me to ponder the uncertain trajectory of my future—a path sometimes beyond our own control (M_4_, 24 years old, Zhejiang).’*

As interviewees expressed, amassing wealth entails continual effort. Driven by the pursuit of improved financial standing, they find themselves with limited time to spend with their children. Despite experiencing this regrettable reality in their upbringing, they remain overwhelmed managing both work and childcare responsibilities.

## 5 Discussion

To shed light on gender roles in childcare into specifical perspective, this paper is based on 25 Chinese immigrants who resided in Portugal over 3 years. Drawing from global literature on gender inequality and traditional Confucian values, the study contextualizes these perspectives within the unique setting of migration. The sample is demographically diverse in terms of age, marital status, education, income and geographical origin. Data analysis shows that Chinese immigrant women are less likely to rely on traditional cultural norms, while men’s involvement in caring is strongly associated with their level of education.

In addition, the data analysis shows that regardless of age variation, male migrants with lower educational attainment from Zhejiang, China, seem more easily influenced by the gender perception where giving birth is a priority task of women and they should assume the care responsibilities. Male immigrants who obtained bachelor’s degree or above have a more egalitarian gender belief. Even though being single, some men have expressed their willingness toward women staying at home of caring for children. Regional culture of origin seems to foster a strong cultural impact on people’s choice. In the case of women, on the other hand, they all agree that being confined solely to family tasks is detrimental not only to their ability to interact socially, but also to their mental well-being. It refers to the spousal relationship dynamics as well. This echoes the research that women with dependent children lean toward a part-time job and other reduced working-hour position (Houston, 2005). Taking part into workplace helps to build a firm familial position compared to their working partner. Women’s awareness of self-worth may attribute to the feminism progressed in China since *Reform and Opening-up* in 1978.

Similarly, younger Chinese immigrants expose a more open-minded approach to dividing household responsibilities, particularly when it comes to childcare. The trend can be explained that second generation immigrants reported less traditional gender roles than first-generational immigrants (Van De Vijver, 2007). Both men and women acknowledge the pivotal role of mutual respect in shaping how household tasks are managed. Furthermore, there is a noticeable shift away from the traditional expectation of mothers-in-law dictating cleaning duties to their daughters-in-law among migrant families.

The other point to emphasize is the difference in parental involvement according to gender, as women and men take on different caring responsibilities. The results showed that mothers usually are the ones who take primary responsibilities for children’s education and daily care, while fathers are the breadwinners. However, this division does not imply a lack of active involvement from fathers in child-rearing. Rather, it reflects a shared understanding achieved through effective communication and the utilization of each family member’s strengths. In effect, Chinese immigrants engage closely to children’s upbring, this intensive parenting is derived from the one child policy implemented in the early 1990s, making Chinese families become more child-centered (Liu and Lummaa, 2019). Both parents play crucial roles in providing financial support, education background, and personal skills necessary for raising children effectively.

However, the data also show that language barriers and financial instability were often barriers to deeper involvement. On the one hand, learning an additional language can place an extra burden on individuals, often falling on women and exacerbating gender inequality. This can also impact parental-child relationship as a lack of prompt and appropriate conversation. On the other hand, occupational characteristics, mainly referring to the Chinese community from Wenzhou, where both spouses are unable to spare more time to be involved in children’s upbringing, have led to a weaker trend in gender differences between men and women.

Overall, this research, which focuses on Chinese migrants in Portugal, highlights gender disparities in childcare—an area that has been underexplored in scholarly discourse. By examining these dynamics, the study significantly contributes to the understanding of Chinese family structures and filial culture within the European context. It enriches the discourse on the evolution of filial piety beyond China, providing valuable insights into Asian family dynamics in a transnational setting. This research serves as a critical foundation for future studies aimed at exploring transnational families and migrant communities, offering a nuanced perspective on how cultural practices on gender roles adapt and evolve in new environments.

## 6 Conclusion

In contemporary western society, a successful woman is defined to laden with expectations such as nurturing behavior, physical attractiveness, and passivity (Toller et al., 2004). Thus, this definition conduces additional encumbrance to working women. Immigrants navigating global mobility often maintain strong ties to their original culture, influencing their perspectives on gender and childcare practices. Modernization accelerates the changes of family structure and the shift of gender perception on domestic work. Within Chinese migrant families in Portugal, the exact word filial piety “xiao (孝)” is rarely known among young generation immigrants, however, it does not mean the erosion of filial piety. In effect, Chinese migrants still view filial piety as the dominant conception for maintaining family bond, providing filial obligation to aging parents makes a human being “qualified.” Although filial piety is hardly heard within family, the younger generation embodies its essence by demonstrating respect, care, and emotional support for their elders. This cultural norm is evident in their actions, such as entering marriage at a specific age. In terms of mate selection, older migrant parents lean toward partners who embody filial attitudes, emphasizing the importance of marrying early. Such unions fulfill parental expectations by showcasing an understanding of traditional Chinese values, ensuring seamless communication within the family without language barriers. Chinese immigrants residing in Portugal encounter unique gender dynamics compared to those in other Western nations. While, surprisingly, migrant men and women in Portugal, particularly men those from Zhejiang province, hold different attitudes toward household labor and childcare. In general, educational level determines the gender attitudes among Chinese migrants. Highly educated immigrants endeavor to alleviate the gender conflicts by means of resultful communications and respect, seeking to realize familial harmony among couples as well as parents and children, that corresponds to the core value of Confucian culture. By delving into the attitudes of men and women toward gender norms in childcare, this study offers valuable insights into the complexities of immigrant experiences.

While shedding light on these dynamics, the study also reveals its limitations. Notably, a lack of detailed exploration into how local Portuguese cultural influences impact migrants’ engagement with childcare practices represents a significant gap in the research. This omission could affect the understanding of how cross-cultural interactions shape the evolving gender roles within these families. Future studies should aim to address this by delving deeper into the nuances of these cultural exchanges, exploring how integration into Portuguese society may modify or reinforce traditional childcare practices and gender expectations within the migrant community. Furthermore, the study’s reliance on a limited number of participants introduces potential biases and constraints in capturing the full range of experiences among Chinese migrant families. This limitation suggests the necessity for larger, more diverse sample sizes in future research endeavors. By expanding the participant pool, researchers can capture a wider array of perspectives, including variations across different socioeconomic backgrounds, regions of origin, and lengths of stay in Portugal. This broader scope will not only enrich the findings but also enhance the generalizability of the results, allowing for a more nuanced understanding of the complexities involved in how gender roles and childcare practices are negotiated in a transnational context.

In conclusion, this study underscores the importance of reevaluating gender norms in childcare within the context of Chinese migrant families in Portugal, highlighting the complexities of cultural influences, evolving gender roles, and the quest for familial harmony. As immigrants navigate the intersection of tradition and modernity, continued research in this area is crucial for fostering inclusivity, understanding, and support within multicultural societies.

## Data Availability

The raw data supporting the conclusions of this article will be made available by the authors, without undue reservation.

## Footnotes

^1^It is important to note the term ‘local Chinese’ refers to individuals who run their own business, including bars, restaurants, retail shops, and supermarkets. Furthermore, they or their children were either born in Portugal or migrated there at a very young age.

^2^Compared to the mentioned above ‘local Chinese’, newcomers stand for the certain group of immigrants who work in other than catering and retail industries. Duration of staying is not applied to the division of these two groups.

## References

[ref1] Bartram D. Poros M. Monforte P. (2014). Key concepts in migration. New Delhi: Sage Publication.

[ref2] Bauman Z. (2000). Liquid modernity. Cambridge: Polity Press.

[ref3] Beck U. Beck-Gernsheim E. (2002). Individualization: Institutionalized individualism and its social and political consequences. London: Sage Publications.

[ref4] Beck U. Giddens A. Lash S. (1994). Reflexive modernization: Politics, tradition, and aesthetics in the modern social order. Stanford: Stanford University Press.

[ref5] Bedford O. Yeh K. H. (2021). Evolution of the conceptualization of filial piety in the global context: from skin to skeleton. Front. Psychol. 12, 1–14. doi: 10.3389/fpsyg.2021.570547, PMID: 33854455 PMC8039149

[ref6] Bianchi S. M. Milkie M. A. Sayer L. C. Robinson J. P. (2000). Is anyone doing the housework? Trends in the gender division of household labor. Soc. Forces 79, 191–228. doi: 10.2307/2675569

[ref7] Blair S. L. Madigan T. J. (2019). Dating, marriage, and parental approval: an examination of young adults in China. Soc. Sci. Q. 100, 2351–2368. doi: 10.1111/ssqu.12718

[ref8] Blossfeld H. P. Kulic N. Skopek J. Triventi M. (2017). Childcare, early education and social inequality: An international perspective. Cheltenham: Edward Elgar Publishing.

[ref9] Bola Elegbede C. Adebayo Abidogun M. (2023). “Women’s experience of balancing work and family roles: counselling strategies for promoting work-life balance.” *10th International Congress on Humanities and Social Science in a Changing World*. 1–10.

[ref10] Bornstein M. H. (2017). Parenting in acculturation: two contemporary research designs and what they tell us. Curr. Opin. Psychol. 15, 195–200. doi: 10.1016/j.copsyc.2017.03.020, PMID: 28813262 PMC5604855

[ref11] Brinton M. C. Oh E. (2019). Babies, work, or both? Highly educated women’s employment and fertility in East Asia. Am. J. Sociol. 125, 105–140. doi: 10.1086/704369

[ref12] Bulanda R. E. (2004). Paternal involvement with children: the influence of gender ideologies. J. Marriage Fam. 66, 40–45. doi: 10.1111/j.0022-2455.2004.00003.x

[ref13] Buttiler M. B. Zhou Q. Uchikoshi Y. (2023). Reasons for migration, parental acculturation, and language: the case of Chinese American and Mexican American parents and dual language learners. Front. Psychol. 14, 1–12. doi: 10.3389/fpsyg.2023.1237143, PMID: 37744593 PMC10513063

[ref14] Cano T. Hofmeister H. (2022). The intergenerational transmission of gender: paternal influences on Children’s gender attitudes. J. Marriage Fam. 85, 193–214. doi: 10.1111/jomf.12863

[ref15] Chan W. Ho Y. Leung Y. Chochinov M. Neimeyer A. Pang C.. (2012). The blessings and the curses of filial piety on dignity at the end of life: lived experience of Hong Kong Chinese adult children caregivers. J. Ethn. Cult. Divers. Soc. Work. 21, 277–296. doi: 10.1080/15313204.2012.729177

[ref16] Charlton B. Andras P. (2003). The modernization imperative. Exeter, UK: Imprint Academic.

[ref17] Charmaz K. (2014). Constructing grounded theory: A practical guide through qualitative analysis. 2nd Edn. Thousand Oaks: Sage Publication.

[ref18] Chen W. Shi L. Luo X. L. (2012). A separation system for China’s mainland: concept and social reality. Int. J. Law Policy Fam. 26, 88–101. doi: 10.1093/lawfam/ebr020

[ref19] Chow N. (2006). The practice of filial piety and its impact on long-term care policies for elderly people in Asian Chinese communities. Asian J. Gerontol. Geriatrics. 1, 31–35.

[ref20] Clark S. C. (2000). Work—family border theory: a new theory of work/family balance. Hum. Relat. 53, 747–770. doi: 10.1177/0018726700536001

[ref21] Cook S. Dong X. (2011). Harsh choices: Chinese Women’s paid work and unpaid care responsibilities under economic reform. Dev. Chang. 42, 947–965. doi: 10.1111/j.1467-7660.2011.01721.x, PMID: 22164881

[ref22] Craig L. (2006). Does father care man fathers share? A comparison of how mothers and fathers in intact families spend time with children. Gend. Soc. 20, 259–281. doi: 10.1177/0891243205285212

[ref23] Czaika M. Reinprecht C. (2022). “Migration drivers: why do people migrate?” in Introduction to migration studies. ed. Scholten P. (Cham, Switzerland: Springer), 49–82.

[ref24] Da W. W. (2003). Transnational Grandparenting: child care arrangements among migrants from the People’s republic of China to Australia. Int. Migration & Integration. 4, 79–103. doi: 10.1007/s12134-003-1020-4

[ref25] Deterding N. M. Waters M. C. (2021). Flexible coding of in-depth interviews: a twenty-first-century approach. Sociol. Methods Res. 50, 708–739. doi: 10.1177/0049124118799377

[ref26] Dioale W. Seda A. (2001). Modernization as changes in cultural complexity: new cross-cultural measurements. Cross-Cult. Res. 35, 129–153.

[ref27] Dion K. L. Dion K. K. (1996). “Chinese adaptation to foreign cultures” in The handbook of Chinese psychology. ed. Bond M. H. (Oxford: Oxford University Press), 457–478.

[ref28] Du F. Dong X. Zhang Y. (2019). Grandparent-provided childcare and labor force participation of mothers with preschool children in urban China. China Popul. Dev. Stud. 2, 347–368. doi: 10.1007/s42379-018-00020-3

[ref29] Ebrey P. B. (2003). Women and the family in Chinese history. London and New York: Routledge.

[ref30] Flick U. (2014). An introduction to qualitative research. 5th Edn. Thousand Oaks: Sage Publications.

[ref31] Florian S. Flippen C. Parrado E. (2022). The labor force trajectories of immigrant women in the United States: interesting individual and gendered cohort characteristics. Int. Migr. Rev. 57, 95–127.

[ref32] Fox B. (2009). When couples become parents: The creation of gender in the transition to parenthood. Toronto: University of Toronto Press.

[ref33] Frone M. R. (2000). Work-family conflict and employee psychiatric disorders: the National Comorbidity Survey. J. Appl. Psychol. 85, 888–895. doi: 10.1037/0021-9010.85.6.888 11155895

[ref34] Frone M. Russel M. Cooper M. (1997). Relation of work-family conflict to health outcomes: a four-year longitudinal study of employed parents. J. Occup. Organ. Psychol. 70, 325–335. doi: 10.1111/j.2044-8325.1997.tb00652.x

[ref35] Gao R. Sacchetto D. (2023). Constructing a transnational childcare bricolage: Chinese migrant families in Italy coordinating transnational mobility and childcare. Eur. Soc. 26, 880–907. doi: 10.1080/14616696.2023.2267638

[ref36] Giddens A. (1991). Modernity and self-identity: Self and society in the late modern age. Stanford: Stanford University Press.

[ref37] Giddens A. (1992). The transformation of intimacy: Sexuality, love and eroticism in modern societies. Cambridge: Polity Press.

[ref38] Gorodzeisky A. Semyonov M. (2017). Labor force participation, unemployment and occupational attainment among immigrants in west European countries. PLoS One 12:e0176856. doi: 10.1371/journal.pone.0176856 28475632 PMC5419508

[ref39] Gray D. E. (2013). Doing research in the real world. London: Sage publications.

[ref40] Gregory A. Milner S. (2009). Editorial: work-life balance: a matter of choice? Gend. Work. Organ. 16, 1–13. doi: 10.1111/j.1468-0432.2008.00429.x

[ref41] Grzywacz J. G. Bas B. L. (2003). Work, family, and mental health: testing different models of work-family fit. J. Marriage Fam. 65, 248–261. doi: 10.1111/j.1741-3737.2003.00248.x

[ref42] Grzywacz J. G. Carlson D. S. (2007). Conceptualizing work—family balance: implications for practice and research. Adv. Dev. Hum. Resour. 9, 455–471. doi: 10.1177/1523422307305487

[ref43] Guo D. (2010). 韩国“孝”伦理思想的历史探源及其当代社会影响. J. Chongqing University of Technol. (Social Sci.). 24, 80–83.

[ref44] Harrison L. E. Huntington S. P. (2000). Culture matters: How values shape human Progress. New York: Basic Books.

[ref45] Haviland W. A. (2002). Cultural anthropology. USA: Harcourt Collage Publishers.

[ref46] He H. Usami S. Rikimaru Y. Jiang L. (2021). Cultural roots of parenting: mothers’ parental social cognitions and Practices from Western US and Shanghai/China. Front. Psychol. 12, 1–11. doi: 10.3389/fpsyg.2021.565040, PMID: 33927660 PMC8076594

[ref47] Heers M. Szalma I. (2022). Gender role attitudes and father practices as predictors of non-resident father-child contact. PLoS One 17, 1–22. doi: 10.1371/journal.pone.0266801, PMID: 35446882 PMC9022857

[ref48] Ho C. (2006). Migration as feminisation? Chinese women’s experiences of work and family in Australia. J. Ethn. Migr. Stud. 32, 497–514. doi: 10.1080/13691830600555053

[ref49] Houston D. (2005). Work–life balance in the twenty-first century. Basingstoke/ New York: Palgrave.

[ref50] Huang C. Y. Lamb M. E. (2015). Acculturation and parenting in first-generation Chinese immigrants in the United Kingdom. J. Cross-Cult. Psychol. 46, 150–167. doi: 10.1177/0022022114555763

[ref51] Kvale S. (1996). Interviews: An introduction to qualitative research interviewing. Thousand Oaks: Sage Publications.

[ref52] Lain L. (1994). “Search for safe haven: the migration and settlement of Hong Kong Chinese immigrants in Toronto” in Reluctant exiles? Migrants from Hong Kong and the new overseas Chinese. ed. Skeldon R. (Armonk, NY: Sharpe), 163–179.

[ref53] Lareau A. (2011). Unequal childhoods: Class, race, and family life. California: University of California Press.

[ref54] Li J. (2004). Gender inequality, family planning, and maternal and childcare in a rural Chinese county. Soc. Sci. Med. 59, 695–708. doi: 10.1016/j.socscimed.2003.11.041, PMID: 15177828

[ref55] Lister R. (2006). Children (but not women) first: new labor, child welfare and gender. Crit. Soc. Policy 26, 315–335. doi: 10.1177/0261018306062588

[ref56] Liu J. Lummaa V. (2019). Whether to have a second child or not? An integrative approach to women’s reproductive decision-making in current China. Evol. Hum. Behav. 40, 194–203. doi: 10.1016/j.evolhumbehav.2018.11.004

[ref57] Liu Y. Wang S. (2020). Chinese immigrants in Europe: Image, Identity and Social Participation. Berlin, Boston: De Gruyter.

[ref58] Major V. Klein K. Ehrhart M. (2002). Work time, work interference with family, and psychological distress. J. Appl. Psychol. 87, 427–436. doi: 10.1037/0021-9010.87.3.427 12090600

[ref59] Mason J. (2002). Qualitative researching. 2nd Edn. Thousand Oaks: Sage Publications.

[ref60] Mathur I. (2023). Confucian social legitimacy: A gendered approach to concepts of filial piety and the order of patriarchal principles Ishita Mathur. Institute of Chine Studies. Available at: https://www.icsin.org/uploads/2023/04/06/51de30e53909c0d720fde13bcff2dd58.pdf (Accessed October 02, 2024).

[ref61] Maurer-Fazio M. Rawski T. Zhang W. (1999). Inequality in the rewards for holding up half the sky: gender wage gaps in China’s urban labor markets, 1988 to 1994. China J. 41, 55–88. doi: 10.2307/2667587

[ref62] McDonald P. Brown K. Bradley L. (2005). Explanations for the provision-utilisation gap in work–life policy. Women Manag. Rev. 20, 37–55. doi: 10.1108/09649420510579568

[ref63] Meares C. (2010). A fine balance: women, work and skilled migration. Women's Stud. Int. Forum 33, 473–481. doi: 10.1016/j.wsif.2010.06.001

[ref64] Meteyer K. Perry-Jenkins M. (2010). Father involvement among working-class dual-earner couples. Fathering 8, 379–403. doi: 10.3149/fth.0803.379

[ref65] Miao C. (2015). The evolution of filial piety in ancient China and its influence on neighboring countries: taking the classic of filial piety as the chief source. Asian Soc. Sci. 11, 319–325. doi: 10.5539/ass.v11n12p319

[ref66] Miles M. B. Huberman A. M. (1994). Qualitative data analysis: An expanded sourcebook. 2nd Edn. Thousand Oaks, CA: Sage Publications.

[ref67] Ni C. (2020). “A comparative study of filial piety and endowment patterns between China and the west.” *3rd international conference on advances in management science and engineering*. Advances in economics, business and management research. 133, 129–134.

[ref68] O’Callaghan C. Kearns R. Woodland L. Dharmagesan G. Harris-Roxas B. (2023). Extended caregiving arrangements in families from Chinese backgrounds: a qualitative research study from Sydney. Australia. Children Youth Services Rev. 145:106795. doi: 10.1016/j.childyouth.2022.106795

[ref69] Park Y. H. (2010). Reflection on Confucian culture and contemporary Korean society. J. Zhejiang University (Human. Social Sci.). 40, 66–76.

[ref70] Plowman L. Mcpake J. Stephen C. (2010). The Technologization of childhood? Young children and Technology in the Home. Child. Soc. 24, 63–74. doi: 10.1111/j.1099-0860.2008.00180.x

[ref71] Pochinga O. (2004). Chinese youth: attitude toward family and marriage. Far Eastern Affairs. 32, 133–149.

[ref72] Polanen M. Colonnesi C. Tavecchio L. Blokhuis S. Fukkink R. (2017). Men and women in childcare: a study of caregiver–child interactions. Eur. Early Child. Educ. Res. J. 25, 412–424. doi: 10.1080/1350293X.2017.1308165

[ref73] Poortman A.-R. Van Der Lippe T. (2009). Attitudes toward housework and childcare and the gendered division of labor. J. Marriage Fam. 71, 526–541. doi: 10.1111/j.1741-3737.2009.00617.x

[ref74] Puar J. (2012). Precarity talk: a virtual roundtable with Lauren Berlant, Judith Butler, Bojana Cvejić, Isabell Lorey, Jasbir Puar, Ana Vujanović. TDR/The Drama Rev. 56, 163–177. doi: 10.1162/DRAM_a_00221

[ref75] Qi X. (2014). Filial obligation in contemporary China: evolution of the culture-system. J. Theory Soc. Behav. 45, 141–161.

[ref76] Röder A. Mühlau P. (2014). Are they acculturating? Europe’s immigrants and gender egalitarianism. Soc. Forces 92, 899–928. doi: 10.1093/sf/sot126

[ref77] Runyon N. (2021). How women’s workplace perspectives differ in China and the west. Thomson Reuters. Available at: https://www.thomsonreuters.com/en-us/posts/legal/womens-perspectives-legal-china/ (Accessed October 02, 2024).

[ref78] Saldana J. (2009). The coding manual for qualitative research. Thousand Oaks: Sage publications.

[ref79] Shirani F. Henwood K. Coltart C. (2012). Meeting the challenges of intensive parenting culture: gender, risk management and the moral parent. Sociology 46, 25–40. doi: 10.1177/0038038511416169

[ref80] Silverstein M. Bengtson V. L. Litwak E. (2003). “Theoretical approaches to problems of families, aging, and social support in the context of modernization” in The need for theory: Critical approaches to social gerontology. eds. Biggs S. Lowenstein A. Hendricks J. (New York: Baywood, Amityville), 181–199.

[ref81] Simpson R. (2004). Masculinity at work: the experience of men in female-dominated occupations. Work Employ. Soc. 18, 349–368. doi: 10.1177/09500172004042773

[ref82] Solinger D. (1999). Contesting citizenship in urban China: Peasant migrants, the state, and the logic of the market. Berkeley, CA: University of California Press.

[ref83] Sprong S. Skopek J. (2023). Childcare utilisation by migration background: evidence from a nationally representative Irish cohort study. Res. Social Stratification and Mobility 84, 100773–100711. doi: 10.1016/j.rssm.2023.100773

[ref84] Stevens D. Kiger G. Mannon S. (2005). Domestic labor and marital satisfaction: how much or how satisfied? Marriage Fam. Rev. 37, 49–67. doi: 10.1300/J002v37n04_04

[ref85] Su L. (2014). A comparison of contemporary filial piety in rural and non-rural China and Taiwan [Master’s thesis, Brigham Young University]. Scholars Archive.

[ref86] Sun J. Wang X. (2010). Value differences between generations in China: a study in Shanghai. J. Youth Stud. 13, 65–81. doi: 10.1080/13676260903173462

[ref87] Toller P. Suter E. Trautman T. (2004). Gender role identity and attitudes toward feminism. Communication Faculty Publications. 51, 85–90. doi: 10.1023/B:SERS.0000032316.71165.45

[ref88] Van De Vijver F. J. R. (2007). Cultural and gender differences in gender-role beliefs, sharing household task and child-care responsibilities, and well-being among immigrants and majority members in the Netherlands. Sex Roles 57, 813–824. doi: 10.1007/s11199-007-9316-z

[ref89] Verniers C. Bonnot V. Assilaméhou-Kunz Y. (2022). Intensive mothering and the perpetuation of gender inequality: evidence from a mixed methods research. Acta Psychol. 227:103614. doi: 10.1016/j.actpsy.2022.103614, PMID: 35576819

[ref90] Vianello F. A. (2022). Conflicting temporalities and the unsustainability of the Italian model of migrant personal care assistant. Int. Migration n/a(n/ a). 61, 328–340. doi: 10.1111/imig.13016

[ref91] Vincent C. Ball S. (2007). ‘Making up’ the middle-class child: families, activities and class dispositions. Sociology 41, 1061–1077. doi: 10.1177/0038038507082315

[ref92] Wang S. (2019). The role of gender role attitudes and immigrant generation in ethnic minority Women’s labor force participation in Britain. Sex Roles 80, 234–245. doi: 10.1007/s11199-018-0922-8, PMID: 30930525 PMC6404549

[ref93] Wang T. P. (2020). Cultura literati Chinesa: Sobre Identidade, Diáspora e Piedade filial [Bachelor Thesis]. Universidade Federal do Rio de Janeiro.

[ref94] Wang Q. Chiang T. Xiao J. (2024). Attitude toward gender inequality in China. Human. Soc. Sci. Commun. 11, 1–14. doi: 10.1057/s41599-024-02857-1

[ref95] Wang S. Coulter R. (2019). Exploring ethnic and generational differences in gender role attitudes among immigrant populations in Britain: the role of Neighbourhood ethnic composition. Int. Migr. Rev. 53, 1121–1147. doi: 10.1177/0197918318802780

[ref96] Xavier R. E. (2017). Royal Asiatic society Hong Kong branch Luso-Asians and the origins of Macau’s cultural development. J. Royal Asiatic Society Hong Kong Branch. 57, 187–205. doi: 10.2307/90013956

[ref97] Yang Y. (2023). Attitudes toward gender roles in child-rearing and their socioeconomic differentials in contemporary China. Chinese J. Sociol. 9, 598–614. doi: 10.1177/2057150X231207121

[ref98] Yao Y. (2001). Study on family support for older adults in China. Beijing: China Population.

[ref99] Yeates N. (2012). Global care chains: a state-of-the-art review and future directions in care transnationalization research. Global Netw. 12, 135–154. doi: 10.1111/j.1471-0374.2012.00344.x

[ref100] Yeh K. H. Bedford O. (2003). A test of the dual filial piety model. Asian J. Soc. Psychol. 6, 215–228. doi: 10.1046/j.1467-839X.2003.00122.x

[ref101] Yeh K. Yi C. Tsao W. Wan P. (2013). Filial piety in contemporary Chinese societies: a comparative study of Taiwan, Hong Kong, and China. Int. Sociol. 28, 277–296. doi: 10.1177/0268580913484345

[ref102] Zhang Z. (2017). Division of housework in transitional urban China. Chinese Sociolog. Rev. 49, 263–291. doi: 10.1080/21620555.2017.1295809

[ref103] Zhang L. De Brauw A. Rozelle S. (2004). China’s rural labor market development and its gender implications. China Econ. Rev. 15, 230–247. doi: 10.1016/j.chieco.2004.03.003

[ref104] Zhao S. (2020). Gender in families: a comparison of the gendered division of child Care in Rural and Urban China. Child and Youth Care Forum. 49, 511–531. doi: 10.1007/s10566-019-09541-5

[ref105] Zhou Y. (2000). The fall of “the other half of the sky”? Chinese immigrant women in the New York area. Women's Stud. Int. Forum 23, 445–459. doi: 10.1016/S0277-5395(00)00106-0

